# BioDADPep: A Bioinformatics database for anti diabetic peptides

**DOI:** 10.6026/97320630015780

**Published:** 2019-11-13

**Authors:** Susanta Roy, Robindra Teron

**Affiliations:** 1 Department of Life Science and Bioinformatics, Assam University Diphu Campus, Diphu, Karbi Anglong 782 462, India

**Keywords:** Diabetes mellitus (DM), therapeutic protein targets, anti-diabetic peptides, text mining, peptide database, cross reactivity

## Abstract

The increasing number of cases for diabetes worldwide is a concern. Therefore, it is of interest to design therapeutic peptides to overcome side effects caused by the available drugs. It should be noted that data on several known anti-diabetic peptides is available in the literature in an organized manner. Hence, it is of interest to collect, glean and store such data in form of a searchable database supported by RDBMS. Data on anti-diabetic peptides and their related data are collected from the literature using manual search. Data on related peptides from other databases (THPdb, ADP3, LAMP, AHTPDB, AVPdb, BioPepDB, CancerPPD, CPPsite, DRAMP, SATPdb, CAMPR3 and MBPDB) are also included after adequate curation. Thus, we describe the development and utility of BioDADPep, a Bioinformatics database for anti-diabetic peptides. The database has cross-reference for antidiabetic peptides. The database is enabled with a web-based GUI using a simple Google-like search function. Data presented in BioDADPep finds application in the design of an effective anti-diabetic peptide.

## Background

Diabetes mellitus is a pandemic non-communicable chronic autoimmune disease affecting the quality of life (diet and lifestyle).It is estimated that diabetes mellitus will rise to 592 million by the year 2035 [1].Approximately 50% of patients with diabetes mellitus are believed to be unaware of their condition [2]. Diabetes Mellitus may go up from ninth to seventh most important reason of death worldwide by 2030 [3]. Based upon the etiology, diabetes mellitus can be divided into two main types, Type 1, "Juvenile Diabetes Mellitus" (Insulin Dependent Diabetes Mellitus) and Type 2, "adult type" (Non-Insulin Dependent Diabetes Mellitus) [4].Type 1 diabetes (Insulin Dependent Diabetes Mellitus, IDDM or juvenile onset diabetes), occurs when the pancreas do not produce enough insulin due to destruction of pancreatic β-cells mediated by auto reactive T-cells resulting in chronic insulitis [5]. In Type 2, "adult type" (Non-Insulin Dependent Diabetes Mellitus), primary insulin resistance, rather than defective insulin production due to β-cells destruction, seems to be the triggering alteration [6].

BioDADPep is an online database of published data about Type 1 and Type 2 diabetes mellitus peptides, their targets and other related data. A comprehensive visualization of all known antidiabetic peptides is important for peptide classification, modification and design. It is of interest to design different formulations of peptides for improved half-life, immunogenicity, chemical activity, solubility, side effects, toxicity and others [7].It is promising to modify the structure to enhance stability, increase the half-life and improve membrane permeability of the peptide after calculating the binding and selectivity using structural features [8].Several methods like truncations, PEGylation and cyclizations available to improve the properties and bioavailability using peptide mimetic design [8].Bioactive peptides that helps assess symptoms of diabetes are also included in the database [9].

## Methodology

### Database architecture and web interface

Collection and organization of data

### Primary data:

**Figure 1:**
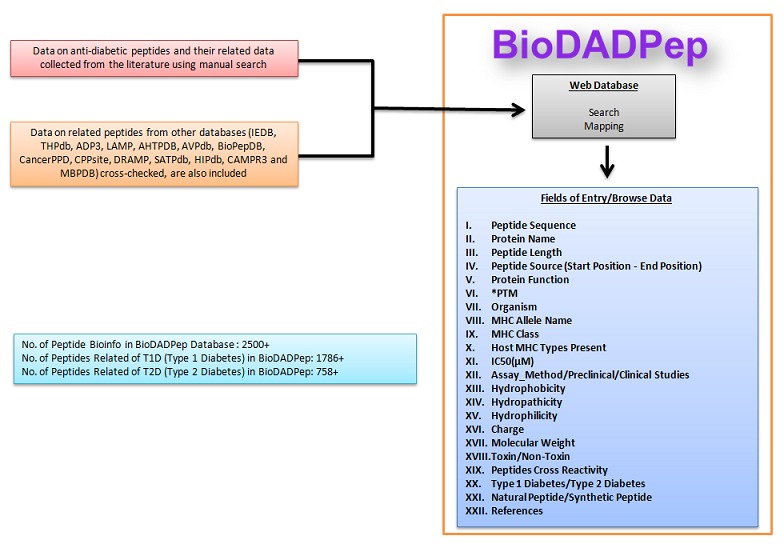
BioDADPep database Schema

Peptides and related data were collected using literature search. We used PubMed to search for such peptides in research and review articles. Peptides were collected based on Type 1 and Type 2 diabetes annotations in literature. References are collected and stored in BioDADPep(Figure 1). This data forms primary data for the database. The source (synthetic or natural) of the peptides is also included in the database. Similar or linked or related peptides from other databases also included in the database.

### Derived data:

Hydrophobicity, hydropathicity, hydrophilicity, charge, molecular weight and toxicity are derived from TOXINPRED [10]. Peptides in BioDADPep are compared with peptides in IEDB (Immune Epitope Database) [11] to extract antidiabetic peptides with cross reactivity features with anticancer, anti-inflammatory, anti-microbial, antioxidant, autoimmune and allergic. Such peptides in databases like THPdb [12], ADP3 [13]; LAMP [14]; AHTPDB [15]; AVPdb [16];BioPepDB [17]; CancerPPD [18]; CPPsite [19]; DRAMP 2.0 [20]; SATPdb [21]; HIPdb [22]; CAMPR3 [23] and MBPDB [24] are also used for enrichment of the database.

### Data retrieval search tool

### User interface

All primary and derived data are included in BioDADPep in respective columns. The data related to a peptide can be browsed using the following parameters (i) accession number, (ii) peptide sequence, (iii) protein name, (iv) peptide length, (v) peptide source (start position-end position), (vi) protein function, (vii) *ptm, (viii) organism, (ix) mhc allele name, (x) mhc class, (xi) host mhc types present, (xii) ic50(µm), (xiii) assay_method/preclinical/clinical studies, (xiv) hydrophobicity, (xv) hydropathicity, (xvi) hydrophilicity, (xvii) charge, (xviii) molecular weight, (xix) toxin/non-toxin, (xx) peptides cross reactivity, (xxi) type 1 diabetes/type 2 diabetes (xxii) natural peptide/synthetic peptide and (xxii) references.

### Browsing:

BioDADPep interface has the following features

(1) Home Page

(2) BioDADPep Search

(3) Data Statistics

(4) Acknowledgment

(5) Help

(6) Contact

### Home page:

**Figure 2:**
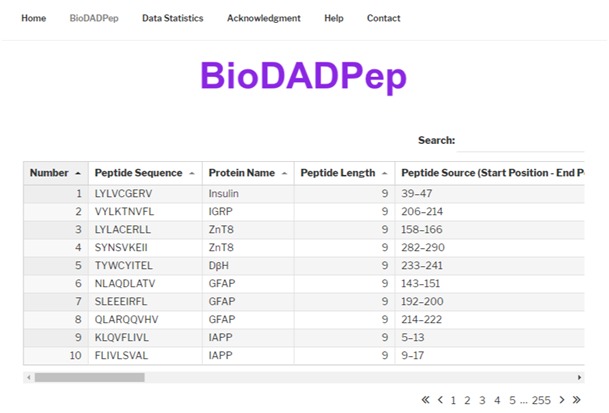
BioDADPep peptide search results

Homepage give introduction on BioDADPep(Figure 2)

### BioDADPep Search:

The BioDADPep database has search utility for keywords.

### Data statistics:

Data statistics with graphs, figures and facts is made available.

### Help:

The HELP page assists to navigate BioDADPep in a step-by-step manner.

## Availability: 

http://omicsbase.com/BioDADPep/
